# Comment on “The effectiveness of home versus community-based weight control programmes initiated soon after breast cancer diagnosis: a randomised controlled trial”

**DOI:** 10.1038/s41416-019-0714-0

**Published:** 2020-01-14

**Authors:** Jennifer R. Bail, Karina I. Halilova, Wendy Demark-Wahnefried, Marina M. Reeves

**Affiliations:** 1 Department of Nutrition Sciences, University of Alabama at Birmingham (UAB), Webb 601, 1675 University Boulevard, Birmingham, AL 35294-3300 USA; 2 Division of Preventive Medicine, UAB, Birmingham, AL USA; 3 School of Public Health, The University of Queensland, Herston, QLD Australia

**Keywords:** Breast cancer, Cancer metabolism

We appreciate Harvie and colleagues’^1^ contribution from the Breast-Activity and Healthy Eating After Diagnosis (B-AHEAD) study to the growing evidence base for weight management programmes among breast cancer survivors. To date, most studies have targeted weight loss following treatment completion,^2,3^ with a small number targeting weight gain prevention during active treatment.^4^ These latter trials show that roughly half are successful in achieving weight maintenance or loss. There is no clear consensus on when to intervene and whether interventions should focus on weight gain prevention or weight loss.

Harvie and colleagues’^1^ decision to intervene soon after surgery provides a valid contribution to the evidence base; however, their decision to target both weight gain prevention and weight loss in those receiving chemotherapy and those remaining chemo-naive and to analyse pooled data as the primary outcome is an interesting approach. Thus the interpretation of these findings is unclear. In the spirit of moving the field forward and to inform future interventions in this area, we seek clarification on some specific issues.

First, B-AHEAD appears to be the first trial that intentionally combines weight gain prevention with weight loss, including breast cancer survivors who are of normal weight, overweight, and obese. Given that strategies and intervention messaging regarding weight gain prevention would differ from weight loss, we are curious on how this was handled during intervention delivery, particularly for the “community” group-based programme. The extant literature suggests that weight management may be more effective if individuals of like weight status are grouped,^5^ which may be particularly true among those with higher degrees of obesity.^6^ We seek clarification on whether the community groups were coordinated based on weight status and intervention messaging, and if not, how this was managed and received by participants in the community arm.

Second, we had concerns regarding data analyses and reporting. We are curious about the decision to use “last observation carried forward (LOCF)”, especially since multiple imputation is the recommended method for missing data in weight loss randomised controlled trials in order to avoid the over-inflation of weight loss that comes from LOCF.^7^ Did the authors conduct sensitivity analyses to test assumptions around missing data? Moreover, because the goals of weight control versus weight loss are different in relation to the effect on the primary outcome of weight change, the reporting of weight change for the combined sample as the primary outcome is questionable. Although the study is largely underpowered for the subgroup analyses, interpretation of these findings is more useful.

Third, we were surprised by the data collection timepoints of baseline, 6 months, and 12 months and were curious as to the rationale for the absence of collection at 12 weeks to coincide with end of intervention. A 12-week timepoint may have aided in understanding the pattern of weight change, particularly since substantial recidivism may have occurred from end of intervention to the 6-month data collection and may have obfuscated the results.

Lastly, as noted, the best timing for delivering weight management interventions among cancer survivors is a highly debated topic.^5^ The B-AHEAD study focussed on early delivery within 12 weeks of surgery. However, most eligible women (58%) chose to not participate, which leads us to ask, “is this the right timing?” In their previous article, Harvie et al.^8^ compared B-AHEAD recruitment rates (42% of eligible women) to clinical/treatment trial rates (47%) in their main recruitment site and imply that their recruitment rate was good. Yet, this recruitment rate is much lower than weight management trials that have recruited posttreatment.^9,10^ Hence, it is important to understand the feasibility of uptake depending on the timing, as well as the intended and communicated purpose. We are interested to know how B-AHEAD was presented to women. Was it presented as a weight loss trial? If it had focussed purely on weight gain prevention or if the timing was different, we wonder if uptake may have been higher. Moreover, as neither intervention (home or community) prevented weight gain nor induced weight loss among those receiving chemotherapy, the question remains as to the optimal timing of a weight management intervention.

In summary, we acknowledge the contribution of the Harvie et al. study to the field, including the reporting on cost-effectiveness outcomes. Clarification on the questions posed here will help move the field of energetics and cancer forward and inform future interventions in this area, particularly with respect to optimal timing and targets (weight gain prevention versus weight loss) and the potential impact that these approaches may have on uptake and outcomes.

## Data Availability

Not applicable.
